# Experimental Meningococcal Sepsis in Congenic Transgenic Mice Expressing Human Transferrin

**DOI:** 10.1371/journal.pone.0022210

**Published:** 2011-07-21

**Authors:** Marek Szatanik, Eva Hong, Corinne Ruckly, Morgan Ledroit, Dario Giorgini, Katarzyna Jopek, Marie-Anne Nicola, Ala-Eddine Deghmane, Muhamed-Kheir Taha

**Affiliations:** 1 Unité des Infections Bactérienne Invasives, Institut Pasteur, Paris, France; 2 Imagopole and Plate-Forme d'Imagerie Dynamique, Institut Pasteur, Paris, France; Columbia University, United States of America

## Abstract

Severe meningococcal sepsis is still of high morbidity and mortality. Its management may be improved by an experimental model allowing better understanding of its pathophysiology. We developed an animal model of meningococcal sepsis in transgenic BALB/c mice expressing human transferrin. We studied experimental meningococcal sepsis in congenic transgenic BALB/c mice expressing human transferrin by transcriptional profiling using microarray analysis of blood and brain samples. Genes encoding acute phase proteins, chemokines and cytokines constituted the largest strongly regulated groups. Dynamic bioluminescence imaging further showed high blood bacterial loads that were further enhanced after a primary viral infection by influenza A virus. Moreover, IL-1 receptor–associated kinase–3 (IRAK-3) was induced in infected mice. IRAK-3 is a negative regulator of Toll-dependant signaling and its induction may impair innate immunity and hence result in an immunocompromised state allowing bacterial survival and systemic spread during sepsis. This new approach should enable detailed analysis of the pathophysiology of meningococcal sepsis and its relationships with flu infection.

## Introduction

*Neisseria meningitidis* (Nm) is an exclusively human bacterium that first adheres to the epithelium of the nasopharynx and may then be translocated to invade the bloodstream. Bacteria can then cross the blood brain barrier to invade the subarachnoidal space of the central nervous system. During these phases of invasive infection, bacteria interact with different cellular barriers and inflammatory cells. They also release a large number of bacterial components such as lipooligosaccharide (LOS) and peptidoglyan that in turn contribute to the bacteria-host interaction [1], [2]. The integral analysis of bacteria-host interaction is hindered by the few available relevant animal models due to the restriction of Nm to human [3]. We have recently used transgenic C57B6/SJLJ mice expressing the human transferrin (hTf) as an animal model for invasive meningococcal infections [4]. This model allows the growth of *N. meningitidis* as it provides bacteria with the physiological iron source and should permit a reliable analysis of host-bacteria interactions. This model permitted the analysis of the virulence of an emergent meningococcal strain in France and showed its ability to provoke septicemia in these mice with the induction of cytokine production [5].

Sepsis (combination of systemic inflammatory response syndrome (SIRS) and documented infection) is a leading cause of morbidity and mortality worldwide. Severe sepsis involves acute organ dysfunction and its incidence is estimated at 300 per 100,000 population in the United States, France and Germany [6]–[8]. We aimed to develop an experimental model that contribute to improve the management of severe sepsis by testing new emerging treatments (new antibiotics and immune-modulating therapies) and by the pathophysiological analysis of invasive meningococcal infection (a pathognomonic severe sepsis with high mortality rate) combining phenotypical analysis and profiling of gene transcription in infected hosts.

## Methods

### Bacterial strains

*N. meningitidis* strain LNP24198, is a clinical isolate of serogroup C, serotype 2a and subtype P1.7,1 (PorA VR1 = 7.1 and VR2 = 1). It belongs to the clonal complex ST-11 and was described previously [5]. Bacteria were grown on GCB medium (Difco) containing the supplements described by Kellog et al [9]. *Escherichia coli* strain DH5 [10] was used for cloning and plasmid preparation. Luria-Bertani (LB) was used for culturing *E. coli*. When appropriate, ampicillin was used at final concentration of 100 µg/ml and kanamycin was added at final concentration of 50 µg/ml and 100 µg/ml for *E. coli* and *N. meningitidis*, respectively.

Firefly Luciferase expressing gene was amplified from the vector pSP-luc+NF Fusion (Biolabs) using the T7 and SP6 primers (Table 1). The amplified fragment was then blunt-ended and and sublconed into the *Bam*H1 restriction site of the previously described recombinant plasmid pTE-KM [11]. This site is located between the meningococcal *pilE* gene encoding the pilin (the major subunit of pili) and the *aph-3*′ gene that confers resistance to kanamycin [11]. The resulting recombinant plasmid was named pDG29 and used to transform the strain LNP24198 as previously described [12] to obtain the luciferase expressing strain NM0804.

**Table 1:** Primers used for RT-PCR analysis.

Mouse genes	Forward 5′ - 3′	Revers 5′ - 3′	cDNA	Reference
**Crp**	5′-GGTCATGAAGACATGTTTAAAA-3′	5′-CCAAAGACTGCTTTGCATCA-3′	460 bp	This work
**Saa3**	5′-ACAGCCAAAGATGGGTCCAGTTCA-3′	5′-ATCGCTGATGACTTTAGCAGCCCA-3′	185 bp	MGI3822749
**Irg1**	5′-ACGGCTTGAAAGTGAACCAC-3′	5′-GAGGGTGGAATCACTTTGGT-3′	439 bp	This work
**Lcn2**	5′-GTTCACTCTGGGAAATATGC-3′	5′-CAGGGGACAGCTCCTTGGTTCTTC-3′	167 bp	This work
**IL-6**	5′-TTCCTCTCTGCAAGAGACT-3′	5′-TGTATCTCTCTGAAGGACT-3′	452 bp	MGI507
**Cxcl1**	5′-GCTGGGATTCACCTCAAGAA-3′	5′-TGGGGACACCTTTTAGCATC-3′	169 bp	This work
**IL-1β**	5′-GGCTGCTTCCAAACCTTTGA-3′	5′-GAAGACACGGATTCCATGGT-3′	710 bp	MGI2662453
**Casp4**	5′-TGGGAACTCTGGAGAAATG-3′	5′-AATGAAGTCCTTCTCCACG-3′	122 bp	MGI2668232
**Sele**	5′-TCTGGACCTTTCCAAAATGG-3′	5′-CAAGCTAAAGCCCTCATTGC-3′	249 bp	This work
**Selp**	5′-TCCTGCTACCCAGGCTTCTA-3′	5′-ATCCCAGAAGCCAAACATTG-3′	197 bp	This work
**Irak3**	5′- CGGAAAGTCTTCCCTGTCTG-3′	5′-ACCTCCATGATCACGATTCC-3′	192	This work
**Hprt**	5′-AAGCAGTACAGCCCCAAAAT-3′	5′-CATAGTGCAAATCAAAAGTC-3′	392 bp	[34]

### Mice and experimental infections

BALB/c mice were in-house bred and were kept in a biosafety containment facility, in filter-topped cages with sterile litter, water and food, according to institutional guidelines. Congenic mice were established in our laboratory by 10 backcrossing of male C57BL/6/SJL progenitors containing hTf gene [4] and BALB/c females (Janvier France). At every generation, tail biopsies were collected only females and DNAs were prepared to detect hTf gene by PCR as previously described [4]. RT-PCR analysis also confirmed the expression of the gene after the N3 and the N10 backcrosses.

For intraperitoneal bacterial infection of mice, bacterial inocula, were adjusted to selected density by dilution in phosphate-buffered saline (PBS). Viral respiratory infection (using the mouse-adapted influenza A virus strain, A/Scotland/20/74 (H3N2)) was performed as previously described [4], [13]. Individual animals were used for each time point.

### Cytokine immunoassays

At selected time points, sera were collected from infected mice and stored at −80°c until testing by ELISA (R&D Systems Europe) for the titration of immunoreactive IL-6, TNF-α and KC, according to manufacturer's recommendations.

### Imaging of bioluminescence from animals

D-luciferin potassium salt (Caliper Life Sciences), the substrate of Firefly luciferase, was dissolved in phosphate-buffered saline at a concentration of 30 mg/ml At the appropriate time of infection, 100 µl (3 mg) of luciferin was given to mice by intraperitoneal injection. Mice were then anesthetized using a constant flow of 2.5% isoflurane mixed with oxygen, using an XGI-8 anesthesia induction chamber (Xenogen Corp.). The mice were maintained for at least 5 min so that there was adequate dissemination of the injected substrate. Bacterial infection images were acquired using an IVIS 100 system (Xenogen Corp., Alameda, CA) according to instructions from the manufacturer. Analysis and acquisition were performed using Living Image 3.1 software (Xenogen Corp.). Images were acquired using 5 min of integration time with a binning of 16. All other parameters were held constant. Quantifying was performed using the photons per second emitted by each mouse. When required, quantifying was also performed by defining regions of interest (skull and organs). An uninfected mouse in the same conditions of acquisition was used for subtracting the background. For ex vivo analyses, the mouse was euthanized for removing the organs that were imaged for 10 min after the addition of the substrate.

### Microarray experiments

Animals for RNA preparation (uninfected mice and 6 h-infected mice) were anesthetized by intraperitoneal injection of 300 mg/kg sodium pentobarbital were exsanguinated by intracardiac puncture and blood was washed out from blood vessels by intracrdiac injection of 6 ml of phosphate-buffered saline. Brain was then removed and homogenized. Blood and brain samples were used to prepare total RNA using Qiagen Kits (RNeasy protect animal blood kit for blood samples and RNeasy lipid tissue mini kit for brain samples). RNAs were quantified and 1.5 µg were used for each experimental condition. We sponsored Beckman Coulter Genomics (Morrisville, NC, USA) to perform the gene expression profiling using the Agilent.

Mouse Whole Genome 4×44 k Microarrays. Target labeling, hybridization, washing, scanning and data extraction were lead using established procedures for the analysis of Eukaryotic RNA by Agilent Technologies (www.agilent.com). Each condition (infected and uninfected blood and brain samples) was tested at last by two different and independent experiments (biological replicates). Raw microarray data were analyzed using Z Score transformation [14]. For each spot the median pixel intensity for the local background of each spot was subtracted from the median pixel intensity of each spot. Background subtracted pixel intensities for the infected sample were divided by those for the reference channel (uninfected sample). The resulting ratios were log transformed and normalized to the mean of log ratio of all spots and then expressed as unit of standard deviation (SD) of the SD of log ratio of all spots to obtain the Z score of each spot. Differentially expressed transcripts (DET) were then defined as those that have a log ratio equal, higher or lower than 2 standard deviations above/below the mean (i.e. Z score of at least 2 for unregulated transcripts and Z score lower or equal −2 for downregulated transcripts). The microarray processed data (blood and brain) have been deposited in a MIAME compliant database http://www.ebi.ac.uk/arrayexpress/).

The DETs that were identified were further subjected to Gene Ontology (GO) enrichment analysis to identify gene ontology biological process categories that are over-represented in the set of DETs in blood and brain samples. Gene ontology analysis was performed using the Version 2.0 (Released August 15) 2003 of the **E**xpression **A**nalysis **S**ystematic **E**xplorer (EASE) available on line (http://david.abcc.ncifcrf.gov/) with Database for Annotation, Visualization, and Integrated Discovery (DAVID) defined default values [15]. This analysis clusters genes into biological categories and calculates the probability of over- and under-representation of clustered genes within a particular group, compared with the master list by use of Fisher's exact test. The expression of several DETs were also cofrimred by reverse transcriptase (RT)-PCR assays. Reverse transcription of RNA from blood or brain of infected and unifected mice were first performed by random priming using random hexanucleotides then PCR was performed on cDNA using primers correspondig to the following genes Crp, Saa3, Irg1, Lcn2 , Il-6, Cxcl1, Il-1b, Casp4, Sele, Selp, Irak3 and Hprt that used as a control (see Tables 1 ad 2 for details).

**Table 2:** Major groups of upregulated genes in blood and brain of infected mice.

Gene names/products	Gene symbol	Keywords	Accesion N°	Site	fold change**	Z score
C-reactive protein*	Crp	Acute phase proteins	NM_007768	Brain	1.3	3.1
Calcitonin A	Calca		NM_007587	Brain	1.9	4.7
Calcitonin receptor like	Calcrl		NM_007588	Blood	0.3	2
Mannose binding lectin (A)	Mbl1		NM_010775	Brain	1.7	4.2
Serum amyloid A1	Saa1		NM_009117	Brain	2.4	5.9
Serum amyloid A3*	Saa3		NM_011315	Brain	3.3	8.2
Immunoresponsive gene 1*	irg1		AK152177	Brain	2.1	5.1
Lipocalin 2*	Lcn2		NM_008491	Brain	1.5	3.5
Peptidoglycan recognition protein2	Pglyrp2	Innate immunity	NM_021319	Brain	1.7	4.1
Peptidoglycan recognition protein3	Pglyrp3		NM_207247	Brain	1.1	2.5
Toll-like receptor 4	Tlr4		AK014533	Brain	1.0	2.3
IL-1 receptor–associated kinase–3*	Irak3		NM_028679	Brain	1.3	3.1
interleukin-1 beta*	Il1b	Immune response	NM_008361	Brain	1.3	3
interleukin-6*	Il6		NM_031168	Blood, Brain	2.2	4, 5.3
interleukin-9	Il9		NM_008373	Brain	1.1	2.5
interleukin-19	Il19		NM_001009940	Brain	2.0	4.8
interleukin-23	Il23		NM_031252	Brain	1.1	2.4
Chemokine Cxcl1 (KC)*	Cxcl1		NM_008176	Blood, Brain	2.6	6.4
Chemokine Cxcl2	Cxcl2		NM_009140	Blood, Brain	2.6	6.3
Defensin beta 1	Defb1		NM_007843	Brain	1.3	3.1
Defensin beta 6	Defb6		NM_054074	Brain	1.8	4.4
Defensin beta 7	Defb7		NM_139220	Brain	1.6	3.8
CD80 co-stimulatory molecule)	Cd80		NM_009855	Brain	1.6	3.8
Endothelial cells selectin*	Sele	Cell adhesion	NM_011345	Brain	2.4	5.8
Platelet selectin*	Selp		NM_011347	Brain	2.3	5.6
Claudin 4	Cldn4		NM_009903	Brain	1.0	2.3
Claudin 7	Cldn7		NM_016887	Brain	1.0	2.3
Claudin 17	Cldn17		NM_181490	Brain	1.4	3.4
Claudin 18	Cldn18		NM_019815	Brain	2.2	5.3
Cadherin 2	Cdh2		AK084821	Brain	1.3	3.2
Intercellular adhesion molecule 1	Icam1		NM_010493	Brain	1.0	2.3
Caspase 4*	Casp4	Apoptosis	NM_007609	Brain	1.1	2.5

* Genes that are also analyzed by RT-PCR.

** Log10 of the ratio of intensity of infected/non infected mice after correction for the background.

### Statistical analysis

Data are expressed as the mean ± SD of triplicate samples, and the reproducibility was confirmed at least in three separate experiments. Statistical analysis were performed using student *t* test (two-way Annova) test, and considered significant if *P*<0.05.

### Ethics Statement

This study was carried out in strict accordance with the European Union Directive 2010/63/EU (and its revision 86/609/EEC) on the protection of animals used for scientific purposes. Our laboratory has the administrative authorization for animal experimentation (Permit Number 75-1554) and the protocol was approved by the Institut Pasteur Review Board that is part of in the Regional Committee of Ethics of Animal Experiments of Paris region (Permit Number: 99-174). All surgery was performed under sodium pentobarbital anesthesia, and all efforts were made to minimize suffering.

## Results

### Establishment of congenic mice expressing human transferrin

**Figure 1:**
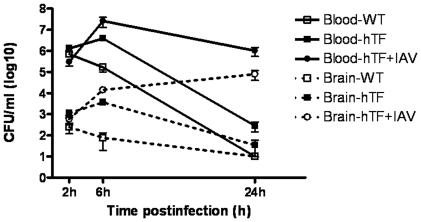
Blood and brain bacterial counts after intraperitoneal challenge. Congenic mice (wild-type, human transferrin expressing transgenic mice with or without IAV infection) were challenged by intraperitoneal injection of 5*10^6^ CFU of the *N. meningitidis* serogroup C strain LNP24198. At each indicated time point, mice were anaesthetized, blood samples were withdrawn and then perfusion was performed for each mouse to wash out blood using phosphate saline buffer. Brains were then extracted. Blood and brain samples were plated to determine the bacterial counts in each fraction. Data represent the means ± SD from 5 independent experiments of groups of five mice per time point in each experiment.

**Figure 2:**
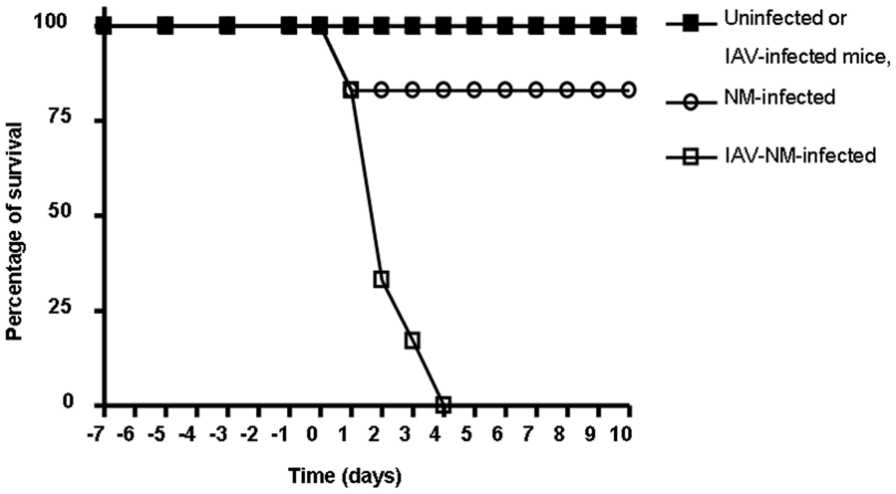
Survival curves of congenic transgenic mice expressing the human transferrin. Two groups of 7 mice were infected by influenza A virus (IAV) by intranasal inhalation. One group of 7 mice inhaled the same volume of phosphate saline buffer. At day 7 of the viral intranasal infection all the mice from the three groups were infected by intraperitoneal injection of 5*106 CFU of the strain 24198. The mice were observed during the whole period up to 10 days after the bacterial secondary challenge. The deaths were scored in each group and the data were expressed as a percentage of survival.

We established a congenic mice expressing hTf gene by performing 10 backcrosses starting by transgenic male C57BL/6/SJL [4] mice with BALB/c females. After each backcross, experimental infection was also performed by intraperitoneal injection of 5*10^6^ CFU of the wild type strain LNP24198. Significant higher bacterial counts in blood and other organs were always observed in congenic transgenic mice compared to their littermate wild-type mice (Fig. 1). The bacterial dose of 5*10^6^ CFU of the wild type strain LNP24198 was sublethal for wild-type mice that recovered after 24 h, whereas 17% mortality of infected congenic transgenic mice were scored after the same period of the bacterial challenge (Fig. 1 and Fig. 2). These data suggest a more severe systemic infection in congenic transgenic mice that can also be mirrored by the peripheral white blood cell (WBC) counts after 6 h of bacterial challenge. Slight but not significant changes were observed in infected wild type compared to uninfected wild type. Lymphocytes decreased while both polymorphonuclear leukocytes and monocytes slightly increased in wild type infected mice after six hours of infections (Table 3). However, total WBC significantly decreased in infected congenic transgenic mice. This decrease was mainly observed for lymphocytes and monocytes that significantly decreased in infected congenic transgenic mice compared to uninfected congenic transgenic mice (Table 3).

**Table 3:** Cell blood formulae in mice.

	Uninfected mice	Infected wild type mice	Infected hTf congenic transgenic mice
Polymorphonuclear cells	890±197	1530±574	610±290
Lymphocytes	4660±1414	2500±1152	1080±210*
Monocytes	250±90	330±150	20±40*
Total WBC	5800±1591	4360±1786	1710±410*

Figures indicate the mean of 6 mice ± standard deviation.

* Significant differences between infected and uninfected mice, *P*<0.05.

The highest levels of proinflammatory cytokines (IL-6, TNF-α and the murine IL-8 homologue KC) in blood and brain of the infected mice were observed after 6 h of infection. These levels were significantly higher in infected congenic transgenic mice than that in infected wild type mice. IL-6 and KC were detected in the brain fraction only in infected congenic transgenic mice after 6 h of infection (Table 4). These data correlate with the results from bacterial counts in blood and brain as well as the blood cell formulae. Our data clearly indicate a combination of meningococcal infection in mice with systemic inflammatory response. The highest biological effects of the experimental infection are observed at 6 h post infection and were significantly more pronounced in congenic transgenic mice expressing human transferrin.

**Table 4:** Levels of cytokines in blood of infected mice.

	wild type mice			hTf transgenic mice		
	2 h	6 h	24 h	2 h	6 h	24 h
Blood						
TNF-α	1191±786	ND	ND	713±691	5642±5657*	ND
IL-6	2174±950	228±322	ND	1171±768	31249±7905*	ND
KC	10975±5631	2589±89	ND	7873±8178	67176±3265*	ND
Brain						
TNF-α	ND	ND	ND	ND	ND	ND
IL-6	ND	ND	ND	ND	ND	ND
KC	ND	753±412	ND	ND	1968±424*	13±18

The tested cytokines were not detectable in uninfected mice. Figures indicate the mean of 6 mice ± standard deviation and (%).

* Significant differences between wild type and transgenic mice, *P*<0.05. ND: non detected.

### Systemic spread of meningococcal infection in mice

**Figure 3:**
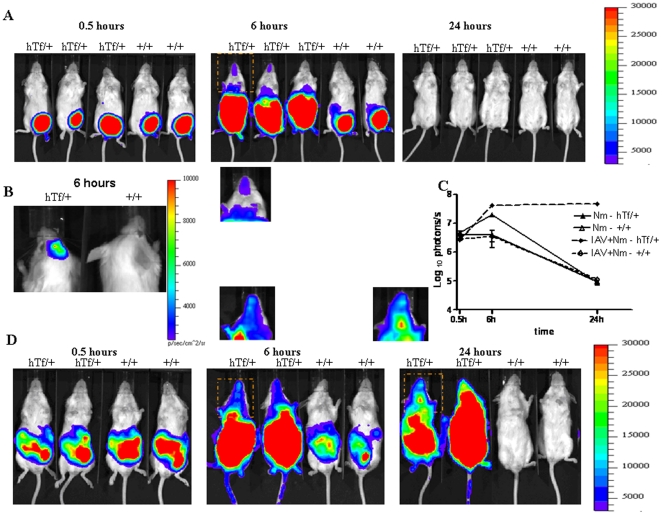
Dissemination of *N. meningitidis* in BALB/c congenic mice that were infected by intraperitoneal injection of 5*10^6^ CFU of *N. meningitidis* (Nm) strain NM0804 expressing the luciferase. Mice were then analyzed for bioluminescence at the indicated times. Images depict photographs overlaid with colour representations of luminescence intensity, measured in photons/second and indicated on the scales, where red is most intense and blue is least intense. (Top row) (A) Ventral views of a three transgenic mice expressing human transferrin (hTf/+) and two wild type mice (+/+) at the indicated times. The framed region of the first transgenic mice is shown below for more details of the bioluminescence imaging in the skull. (B) Dorsal view of the skull of one transgenic mouse expressing human transferrin and one wild type mouse. The substrate of firefly luciferase was injected directly into the lateral ventricle after 6 h of bacterial intraperitoneal challenge. (C) The luminescence of the hTf/+ and (+/+) mice from A and D was quantified and expressed as means ± SD from each category at the indicated times by defining specific representative region of interest encompassing the entire animal.(D) Ventral views of a three transgenic mice expressing human transferrin (hTf/+) and two wild type mice (+/+) at the indicated times. Mice were first infected by influenza A virus (IAV) by intranasal inhalation. Mice were then infected after 7 days by *N. meningitidis* (Nm) strain NM0804 by intraperitoneal injection. The framed region of the first transgenic mice is shown below for more details of the bioluminescence imaging in the skull.

Dynamic bioluminescence imaging approach showed that immediately after the injection of the bacterial suspension, bacteria were mainly present in the peritoneal cavity and start to spread through bloodstream as confirmed by the detection of bioluminescence signal in blood samples. At this early stage of infection, both wild type and congenic transgenic mice showed similar levels of bioluminescence (Fig. 3A and 3C). In wild type mice, bacteria remained mainly in the peritoneal cavity 6 h later with no detectable distal localization of the bioluminescence. At the opposite, bioluminescence was detectable in distal anatomic sites with pronounced signals in the skull (Fig. 3A). When the substrate of firefly luciferase was injected directly into the lateral ventricle after 6 h of bacterial intraperitoneal challenge, only congenic transgenic mice showed signal but not wild type mice (Fig. 3B). This signal is most likely due to the presence of viable bacteria in the brain that can be associated to the endothelial cells of the brain blood vessels and/or in the cerebrospinal fluid in subarachnoidal space. Moreover, bioluminescent signal was significantly higher in congenic transgenic mice compared to the wild type mice as indicated by the total number of photons detected per second (Fig. 3C). After 24 h of infection, no emitted photons were detected (Fig. 3A). These data are in accordance with those obtained from bacterial counts in blood and brain (Fig. 1).

### Host gene expression during experimental infection

We used Agilent gene expression microarray (Whole Mouse Genome (4×44 K) and compared gene expression in infected versus non infected congenic transgenic mice. RNAs were prepared from blood and brain after extensive washing to eliminate circulating blood from vessels. Fold changes were computed and comparison of expression (infected versus uninfected samples) was performed using Z score transformation [14]. Genes that have a Z score exceeding 2 standard deviations above or below the mean represent the most highly or the least expressed genes, respectively. The numbers of differentially expressed transcripts (DET) in the blood of infected mice were 977 and 303 genes that were detected to be upregulated or downregulated, respectively. These numbers were respectively 2364 and 284 in the brain samples of infected mice that include central nervous system cells and cells of walls of blood vessels (mainly endothelial cells).

**Figure 4:**
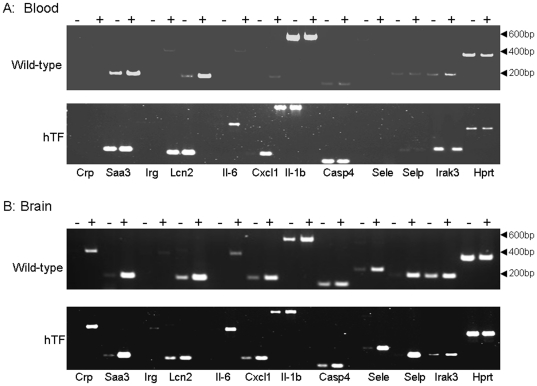
RT-PCR analysis on agarose gel electrophoresis using total RNA extracted from the blood (A) and brain (B) of congenic transgenic and wild type mice expressing human transferrin. RT-PCR experiments for the indicated genes were realized using primers indicated in Table 1. (−) and (+) above the gels indicate uninfected and infected mice respectively. Length markers in bp are shown on the right.

The gene ontology (GO) analysis of the data allowed clustering of the DETs. Relevant GO are indicated in (Table 5) and corresponded to clusters with an enrichment score >1 and a *P* value≤0.05. GO data showed that most of the DETs were related to genes involved in innate immune response to sepsis. In particular, genes involved in acute phase and acute inflammatory responses were upregulated as well as those of the oxygen and reactive oxygen species metabolic process. Downregulated DETs in the blood sample also belonged to innate immune response such as the complement component factor H (Cfh NM_009888 of the GO:0045087∼innate immune response). Factor H is a negative regulator of the alternative pathway of complement. While other positive regulator of the alternative pathway were upregulated such as the complement factor D (Cfd, NM_013459 of the GO:0006954∼inflammatory response). Several DETs such as those involved in acute phase inflammation (Crp, lipocalin2, irg1, serum amyloid 3), cytokines and chemokines expression (IL-6 IL1-ß and Cxcl1), apoptosis (Caspase 4) and cell adhesion molecules selectins E and P were confirmed to be upregulated by RT-PCR (Table 2, Fig. 4). The induction of these genes was not due to the presence of the hTf gene in transgenic mice as non infected wild type and transgenic mice showed the similar signal by RT-PCR (Fig. 4). The DETs were observed in both types of mice although the induction was stronger in transgenic mice such as Crp and IL-6 in the brain fraction (Fig. 4). This higher induction in infected transgenic mice compared to the wild type mice was also observed in cytokines assays in blood and brain fractions (Table 4). It is of interest to note that IL-11 receptor–associated kinase–3 (IRAK-3) is also induced in the brain fraction (Table 2 and Fig. 4).

**Table 5:** Major gene ontology GO groups.

GO Term	Number of DETs	% of DETs in the corresponding Term
Upregulated blood		
GO:0006954∼inflammatory response	11	3
GO:0007155∼cell adhesion	43	5.4
GO:0008092∼cytoskeletal protein binding	34	4.3
GO:0007267∼cell-cell signaling	29	3.7
GO:0044421∼extracellular region part	50	6.3
GO:0005125∼cytokine activity	17	2.1
GO:0045893∼positive regulation of transcription. DNA-dependent	24	3.0
Upregulated brain		
GO:0044421∼extracellular region part	113	6.9
GO:0006952∼defense response	70	4.3
GO:0005125∼cytokine activity	40	2.4
GO:0007186∼G-protein coupled receptor protein signaling pathway	162	9.9
GO:0009617∼response to bacterium	27	1.7
GO:0004175∼endopeptidase activity	53	3.2
GO:0004222∼metalloendopeptidase activity	19	1.2
GO:0006953∼acute-phase response	10	0.6
GO:0019882∼antigen processing and presentation	13	0.8
GO:0002064∼epithelial cell development	6	0.4
GO:0001819∼positive regulation of cytokine production	12	0.7
GO:0050727∼regulation of inflammatory response	11	0.7
GO:0016337∼cell-cell adhesion	25	1.5
GO:0045944∼positive regulation of transcription from RNA polymerase II promoter	39	2.4
GO:0033028∼myeloid cell apoptosis	3	0.2
GO:0030593∼neutrophil chemotaxis	5	0.3
Downregulated blood		
GO:0045087∼innate immune response	6	2.2
GO:0005764∼lysosome	9	3.2
GO:0004672∼protein kinase activity	18	6.5
GO:0001846∼opsonin binding	3	1.1
GO:0050764∼regulation of phagocytosis	4	1.4
GO:0030001∼metal ion transport	14	5.1
Downregulated brain		
GO:0016021∼integral to membrane	73	34.1
GO:0003700∼transcription factor activity	19	8.9
GO:0007186∼G-protein coupled receptor protein signaling pathway	36	16.8
GO:0007166∼cell surface receptor linked signal transduction	41	19.2
GO:0048538∼thymus development	4	1.9
GO:0006955∼immune response	10	4.7
GO:0009408∼response to heat	3	1.4

### Influenza A virus and meningococcal sepsis

We have previously reported an experimental model of sequential IAV-Nm infection in BALB/c mice [13]. This model takes advantage from the observation of the spatiotemporal association between flu epidemics and meningococcal infectious [16], [17]. We therefore tested whether a prior viral infection with influenza A virus (IAV) can also enhance systemic meningococcal infection and increase the level of bacteraemia to allow more effective bacterial localization in the brain of congenic transgenic mice.

**Figure 5:**
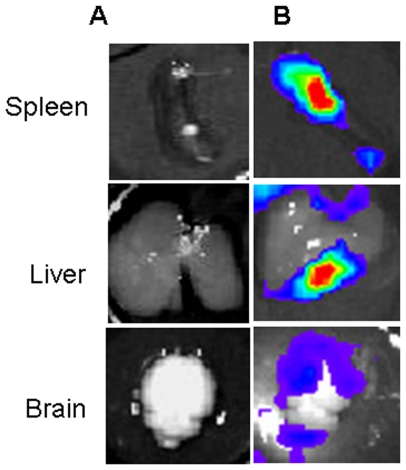
Ex-vivo images of organs from mouse infected by influenza A virus (IAV) intranasal inhalation and by Nm at day 7 by intraperitoneal injection. Mouse was dissected to determine which organs were the sources of bioluminescence. The spleen, liver and brain were hence removed and the mixture containing the substrate of firefly luciferase was added. Dynamic bioluminescence imaging from noninfected (A) or infected organs (B) are shown.

Higher bacterial counts in blood and brain were observed after 6 h and 24 h of infection in IAV-Nm infected congenic transgenic mice than that detected in Nm-infected congenic transgenic mice (Fig. 1). Moreover, higher mortality was also observed in IAV-Nm infected congenic transgenic mice with 83% of death after 24 h and 100% of mortality after three days of the bacterial infection. Mortality level was 17% among Nm-infected congenic transgenic mice after 24 h of bacterial infection and all the other surviving mice recovered from the bacterial infection (Fig. 2). Dynamic imaging showed significant higher signal of bioluminescence in congenic transgenic mice that were first infected by IAV at J-7 before the bacterial intraperitoneal challenge. It is noteworthy that the emission of photons continued to increase at 24 hours of infection with even more pronounced signal in the skull (Fig. 3D).Upon dissection of infected mice, signal were detected in blood, spleen, liver ad brain (Fig. 5). These data further support the association between these two infections: the flu and a secondary meningococcal with the primary intranasal IAV infection that enhances the systemic meningococcal infection by intraperitoneal route. Recent microarray data showed that enriched GO terms in response to IAV infection contained biological process of innate immune response such as GO:0045087 [18]. This GO is also enriched in our microarray analysis (Table 5). It is noteworthy that this GO contains the factor H binding gene that may show the same regulation as the human gene as Nm binds specifically human factor H [19], [20] Indeed, the administration of human fH to infant rats challenged increased significantly the bacterial load in blood [21].

## Discussion

Meningococcal components (pathogen associated molecular patterns, PAMPs) such as the LOS and peptidoglycan are released and are responsible for the induction of systemic inflammatory response. These PAMPS are then recognized by there corresponding pattern recognition molecules such as Toll-like receptors (TLRs), peptidoglycan recognition proteins (PGRP) and Nod family (Nucleotide-binding oligomerization domain containing proteins). Significant induction was observed in the brain samples for TLR4, IRG1, PGRP2 and PGRP3. These data corroborate the apparent tropism to the central nervous system as suggested by the dynamic imaging, the cytokine production and the detection of viable bacteria in the brain samples. Upon recognition of the meningococcal PAMPs, signal is transduced through pathways involving MyD88, IL-1 receptor–associated kinase–1 (IRAK-1) and IRAK-4. Once IRAK complex is phosphorylated it interacts with TRAF6 (TNF –α receptor associated factor 6) that leads to downstream activation of NF-κB and the induction of proinflammatory cytokines and chemokines [22], [23]. Genes of the signal transduction pathways were activated in our transcriptomic profiling analysis of brain samples (Table 5 GO:0007186). Interestingly, our microarray data also show the induction of interleukin-1 receptor-associated kinase 3 (IRAK-3 also known as IRAK-M) in the brain samples. IRAK-3 negatively regulates TLR signaling [24], [25] and may hence impair cell function upon the presence of bacterial PAMPs. IRAK-3 plays a pivotal role in innate immunity that may result in an immunocompromised state during sepsis [26]. After the early proinflammatory response in sepsis, a subsequent antiinflammatory step is suggested to worsen sepsis in part due to sepsis-induced leukocyte “immunoparalysis”. Indeed, fatality was higher in patients with sepsis associated with monocyte deactivation [27]. Moreover, peripheral blood monocytes isolated from septic patients displayed enhanced expression of IRAK-3, as compared with cells isolated from nonseptic controls [28]. Our data on the low number of circulating monocytes and the induction of IRAK-3 are in agreement of this scheme. The absence of IRAK-3 was shown to lead to better clearance of *Pseudomonas aeruginosa*.from the lung and blood in mice [26].

Recruitment and sequestration of blood cells in vessels of the central nervous system may be responsible for pronounced modification of these cells in infected transgenic mice. KC chemokine constantly detected in the brain samples (Table 4) and transcriptomic profiling showed induction of adhesion molecules such as endothelial cells selectin (SelE) and Intercellular adhesion molecule ICAM-1 in the brain samples. Other cell-to-cell adhesion molecules were also induced such as integrins, cadherins and catenins in the brain samples.

Acute phase markers such CRP and the calcitonin genes family as well as those belonging to serum amyloid A and lipocalin families are also induced in the brain samples, suggesting direct interaction of bacteria and bacterial components with cells in these samples. Lipocalin 2 (LCN2) was recently reported as an acute phase protein to be produced at the blood brain barrier by the choroid plexus epithelial cells and the endothelial cells of blood vessels [29]. The expression of serum amyloid 3 (ssa3) was reported to be induced in epithelial cells through TLR4/MyD88/NF-κB Signaling pathway [30]. Our data show that genes encoding acute phase proteins, chemokines and cytokines constituted the largest strongly regulated group.

Induction of apoptosis and/or its modulation is increasingly reported in pathogenic bacteria [31]. We have recently reported that meningococcal strains of the clonal complex ST-11, such as the strain LNP24198 used in the current study, are able to upregulate TNF-α receptor I (TNFRI) in epithelial cells and to induce apoptosis through of TNF-α/TNFRI signaling [32]. Our data confirm this finding as several transcripts corresponding to the tumor necrosis factor receptor superfamily were induced such as the member 10b (Tnfrsf10b) involved in TNF-α-related apoptosis [33].

We presented in this work an extensive approach of characterization of experimental meningococcal sepsis paving the way for preclinical research. Detailed analyses of the functions/roles of differentially expressed genes completed by kinetics of gene expression should allow better understanding of the pathophysiology of this sepsis as well as the analysis of new therapeutic approaches.

## References

[1] Rosenstein NE, Perkins BA, Stephens DS, Popovic T, Hughes JM (2001). Meningococcal disease.. N Engl J Med.

[2] van Deuren M, Brandtzaeg P, van der Meer JW (2000). Update on meningococcal disease with emphasis on pathogenesis and clinical management.. Clin Microbiol Rev.

[3] Johansson L, Rytkonen A, Bergman P, Albiger B, Kallstrom H (2003). CD46 in meningococcal disease.. Science.

[4] Zarantonelli ML, Szatanik M, Giorgini D, Hong E, Huerre M (2007). Transgenic mice expressing human transferrin as a model for meningococcal infection.. Infect Immun.

[5] Deghmane AE, Parent du Chatelet I, Szatanik M, Hong E, Ruckly C (2010). Emergence of new virulent *Neisseria meningitidis* serogroup C sequence type 11 isolates in France.. J Infect Dis.

[6] Angus DC, Linde-Zwirble WT, Lidicker J, Clermont G, Carcillo J (2001). Epidemiology of severe sepsis in the United States: analysis of incidence, outcome, and associated costs of care.. Crit Care Med.

[7] Brun-Buisson C, Meshaka P, Pinton P, Vallet B (2004). EPISEPSIS: a reappraisal of the epidemiology and outcome of severe sepsis in French intensive care units.. Intensive Care Med.

[8] Schuerholz T, Marx G (2008). Management of sepsis.. Minerva Anestesiol.

[9] Kellogg DS, Peacock WL, Deacon WE, Brown L, Pirkle DI (1963). *Neisseria gonorrhoeae*. I. Virulence Genetically Linked to Clonal Variation.. J Bacteriol.

[10] Hanahan D (1983). Studies on transformation of *Escherichia coli* with plasmids.. J Mol Biol.

[11] Taha MK, Morand PC, Pereira Y, Eugene E, Giorgini D (1998). Pilus-mediated adhesion of *Neisseria meningitidis*: the essential role of cell contact-dependent transcriptional upregulation of the PilC1 protein.. Mol Microbiol.

[12] Taha MK, So M, Seifert HS, Billyard E, Marchal C (1988). Pilin expression in *Neisseria gonorrhoeae* is under both positive and negative transcriptional control.. Embo J.

[13] Alonso JM, Guiyoule A, Zarantonelli ML, Ramisse F, Pires R (2003). A model of meningococcal bacteremia after respiratory superinfection in influenza A virus-infected mice.. FEMS Microbiol Lett.

[14] Cheadle C, Vawter MP, Freed WJ, Becker KG (2003). Analysis of microarray data using Z score transformation.. J Mol Diagn.

[15] Hosack DA, Dennis G, Sherman BT, Lane HC, Lempicki RA (2003). Identifying biological themes within lists of genes with EASE.. Genome Biol.

[16] Rameix-Welti MA, Zarantonelli ML, Giorgini D, Ruckly C, Marasescu M (2009). Influenza A virus neuraminidase enhances meningococcal adhesion to epithelial cells through interaction with sialic acid-containing meningococcal capsules.. Infect Immun.

[17] Hubert B, Watier L, Garnerin P, Richardson S (1992). Meningococcal disease and influenza-like syndrome: a new approach to an old question.. J Infect Dis.

[18] Lee SM, Chan RW, Gardy JL, Lo CK, Sihoe AD (2010). Systems-level comparison of host responses induced by pandemic and seasonal influenza A H1N1 viruses in primary human type I-like alveolar epithelial cells in vitro.. Respir Res.

[19] Schneider MC, Prosser BE, Caesar JJ, Kugelberg E, Li S (2009). *Neisseria meningitidis* recruits factor H using protein mimicry of host carbohydrates.. Nature.

[20] Schneider MC, Exley RM, Ram S, Sim RB, Tang CM (2007). Interactions between *Neisseria meningitidis* and the complement system.. Trends Microbiol.

[21] Granoff DM, Welsch JA, Ram S (2009). Binding of complement factor H (fH) to *Neisseria meningitidis* is specific for human fH and inhibits complement activation by rat and rabbit sera.. Infect Immun.

[22] Akira S, Takeda K (2004). Toll-like receptor signalling.. Nat Rev Immunol.

[23] Takeda K, Akira S (2004). Microbial recognition by Toll-like receptors.. J Dermatol Sci.

[24] Kobayashi K, Hernandez LD, Galan JE, Janeway CA, Medzhitov R (2002). IRAK-M is a negative regulator of Toll-like receptor signaling.. Cell.

[25] Liew FY, Xu D, Brint EK, O'Neill LA (2005). Negative regulation of toll-like receptor-mediated immune responses.. Nat Rev Immunol.

[26] Deng JC, Cheng G, Newstead MW, Zeng X, Kobayashi K (2006). Sepsis-induced suppression of lung innate immunity is mediated by IRAK-M.. J Clin Invest.

[27] Munoz C, Carlet J, Fitting C, Misset B, Bleriot JP (1991). Dysregulation of in vitro cytokine production by monocytes during sepsis.. J Clin Invest.

[28] Escoll P, del Fresno C, Garcia L, Valles G, Lendinez MJ (2003). Rapid up-regulation of IRAK-M expression following a second endotoxin challenge in human monocytes and in monocytes isolated from septic patients.. Biochem Biophys Res Commun.

[29] Marques F, Rodrigues AJ, Sousa JC, Coppola G, Geschwind DH (2008). Lipocalin 2 is a choroid plexus acute-phase protein.. J Cereb Blood Flow Metab.

[30] Reigstad CS, Lunden GO, Felin J, Backhed F (2009). Regulation of serum amyloid A3 (SAA3) in mouse colonic epithelium and adipose tissue by the intestinal microbiota.. PLoS One.

[31] Grassme H, Jendrossek V, Gulbins E (2001). Molecular mechanisms of bacteria induced apoptosis.. Apoptosis.

[32] Deghmane AE, Veckerle C, Giorgini D, Hong E, Ruckly C (2009). Differential modulation of TNF-alpha-induced apoptosis by *Neisseria meningitidis*.. PLoS Pathog.

[33] Li P, Jayarama S, Ganesh L, Mordi D, Carr R (2010). Akt-phosphorylated mitogen-activated kinase-activating death domain protein (MADD) inhibits TRAIL-induced apoptosis by blocking Fas-associated death domain (FADD) association with death receptor 4.. J Biol Chem.

[34] Seki M, Higashiyama Y, Tomono K, Yanagihara K, Ohno H (2004). Acute infection with influenza virus enhances susceptibility to fatal pneumonia following *Streptococcus pneumoniae* infection in mice with chronic pulmonary colonization with *Pseudomonas aeruginosa*.. Clin Exp Immunol.

